# Real-World Efficacy and Durability of Faricimab in Aflibercept-Resistant Neovascular Age-Related Macular Degeneration

**DOI:** 10.3390/jcm14155412

**Published:** 2025-08-01

**Authors:** Areum Jeong, Huiyu Liang, Seung Chul Baek, Min Sagong

**Affiliations:** 1 Department of Ophthalmology, Yeungnam University College of Medicine, Daegu 42415, Republic of Korea; heart0610@hanmail.net (A.J.); lianghuiyulhy@163.com (H.L.); ngsgnbk@hanmail.net (S.C.B.); 2 Yeungnam Eye Center, Yeungnam University Hospital, Daegu 42415, Republic of Korea

**Keywords:** faricimab, aflibercept, age-related macular degeneration

## Abstract

**Objectives**: This study aimed to evaluate the 6-month real-world outcomes of switching to faricimab in patients with aflibercept-resistant neovascular age-related macular degeneration (nAMD). **Methods**: A retrospective review was conducted on the eyes of 60 patients with aflibercept-resistant nAMD that were switched to faricimab. Best-corrected visual acuity (BCVA) and optical coherence tomography (OCT) parameters, including central subfield thickness (CST), subfoveal choroidal thickness (SFCT), and both the maximum height and width of pigment epithelial detachment (PED), at baseline and 1, 3, and 6 months after switching were evaluated. The type of PED and retinal fluid were also analyzed. **Results**: The results showed that BCVA remained stable at month 6 (*p* = 0.150), while CST significantly decreased (*p* = 0.020), and SFCT remained unchanged (*p* = 0.072). The maximum PED height significantly decreased (*p* = 0.030), while the maximum PED width did not change (*p* = 0.07). The mean injection interval significantly increased from 6.8 ± 2.4 weeks before switching to 11.2 ± 1.7 weeks after switching (*p* = 0.068). Furthermore, the dry macula rate was 43.3% at month 6. **Conclusions**: Switching to faricimab in aflibercept-resistant nAMD patients showed stable visual outcomes, significant anatomical improvements, and reduced treatment burden over 6 months in real-world clinical settings.

## 1. Introduction

Age-related macular degeneration (AMD) is a leading cause of blindness worldwide, particularly among the elderly population [1,2]. Neovascular age-related macular degeneration (nAMD), a severe subtype of AMD, is characterized by the abnormal proliferation of macular neovascularization (MNV), leading to retinal fluid and vision loss [3,4,5]. This pathological angiogenesis and the associated retinal damage significantly impair central vision and the quality of life. Intravitreal injections of antivascular endothelial growth factor (anti-VEGF) agents have revolutionized the treatment of nAMD by mainly targeting vascular endothelial growth factor A (VEGF-A), thereby reducing retinal and choroidal neovascularization and preventing disease progression [4,5,6,7,8].

Faricimab, a recently approved bispecific antibody, offers a new treatment option for patients with nAMD resistant to other anti-VEGF agents. Faricimab simultaneously targets VEGF-A and angiopoietin-2 (Ang-2), a key mediator of vascular instability and inflammation. By neutralizing these pathways, faricimab not only reduces vascular leakage and neovascular growth but also stabilizes the vasculature [9,10]. Furthermore, clinical trials have demonstrated that faricimab extends treatment intervals compared to aflibercept, thereby alleviating the treatment burden and improving patient compliance [10,11,12].

Pigment epithelial detachment (PED) is a common feature of nAMD. Retinal exudative changes accompanied by shallow PED are indicative of MNV, which can lead to complications such as subretinal fluid (SRF), intraretinal fluid (IRF), subretinal pigment epithelial fluid, and hemorrhage under the retina or retinal pigment epithelium (RPE) [13]. These changes could ultimately cause a decline in visual acuity, and large PEDs associated with MNV might lead to an increased risk of RPE tears.

The adoption of faricimab in clinical practice has been increasing, particularly for patients with treatment-resistant nAMD. Studies have reported promising results regarding its efficacy in improving retinal structure, along with prolonged injection intervals [14,15,16,17,18]. However, although clinical data focused on the efficacy and safety of faricimab are growing, evidence remains limited particularly in the real-world setting regarding its effect on PED in nAMD patients. This gap highlights the need for further investigation to better understand the potential benefits of faricimab, especially its impact on diverse aspects of PED, which play critical roles in visual outcomes.

Therefore, this study aimed to assess the real-world efficacy and durability of faricimab in patients with aflibercept-resistant nAMD, with particular attention to both functional outcomes and anatomical changes including PED.

## 2. Materials and Methods

### 2.1. Ethical Statement

This retrospective study adhered to the tenets of the Declaration of Helsinki and received ethical approval from the Institutional Review Board of Yeungnam University College of Medicine (IRB No. 2025-01-008). Due to the retrospective nature of this study, the requirement for obtaining informed consent was waived by the Institutional Review Board of Yeungnam University College of Medicine.

### 2.2. Participants

This study included patients with aflibercept-resistant nAMD between June 2023 and December 2024. During the pre-switch period, all patients received intravitreal aflibercept at a dose of 2 mg under a pro re nata (PRN) regimen after three loading injections. The minimum treatment interval of aflibercept injections was 4 weeks. Patients were switched to faricimab if they showed persistent SRF or IRF on OCT despite receiving at least three consecutive aflibercept injections at 4–6-week intervals. After switching to faricimab, patients were treated using a PRN regimen with a minimum injection interval of 8 weeks. No mandatory loading doses were administered after switching.

Exclusion criteria included a history of vitreoretinal surgery or photodynamic therapy; coexisting ocular diseases that could confound the evaluation of treatment effectiveness, such as retinal vascular occlusion or diabetic retinopathy; and poor OCT image quality due to factors like severe vitreous hemorrhage or dense cataracts, which could interfere with accurate parameter measurement.

### 2.3. Outcome Measurement

Clinical data, including age, gender, subtype of AMD, treatment history, injection number, and treatment intervals, were collected. All patients underwent detailed ophthalmologic evaluations, including BCVA measurement and spectral domain OCT (Heidelberg Spectralis OCT; Heidelberg Engineering, Heidelberg, Germany), at baseline and during follow-up visits at 1, 3, and 6 months after switching to faricimab. The BCVA measured was converted into the logarithm of the minimum angle of resolution (logMAR).

OCT imaging was performed using a standardized protocol, with macular scans centered on the fovea to assess the presence of SRF, IRF, and PED. Quantitative OCT parameters, including CST, SFCT, maximum PED height, and maximum PED width, were measured. PED height was measured as the vertical distance between the inner surface of the detached RPE and the underlying Bruch’s membrane, while PED width was measured as the horizontal distance at the base of the PED using an OCT caliper tool. PED was defined as an elevation of the RPE with a width of 350 µm or greater and categorized as predominantly serous, predominantly fibrovascular, or fibrovascular only, as described in clinical trials (TENAYA and LUCERNE) [12].

### 2.4. Statistical Analysis

Statistical analyses were performed using SPSS version 27.0 (IBM Corp., Chicago, IL, USA). Continuous variables were expressed as the mean ± standard deviation. Paired *t*-tests were used to evaluate changes in normally distributed data, while the Wilcoxon signed-rank test was applied to non-normally distributed variables. Changes in BCVA, CST, SFCT, and maximum PED dimensions at various time points were analyzed using a repeated measures analysis of variance (ANOVA). Multivariate analysis was performed using binary logistic regression. A *p*-value of <0.05 was considered statistically significant.

## 3. Results

### 3.1. Demographics and Clinical Characteristics

Baseline demographics and clinical characteristics are summarized in Table 1. A total of 60 eyes from 60 patients with aflibercept-resistant nAMD were included in this study, with a mean age of 71.8 ± 9.7 years. The cohort consisted of 60% males and 40% females. Among the patients, 40% (24 patients) were diagnosed with typical nAMD, 53% (32 patients) with polypoidal choroidal vasculopathy (PCV), and 7% (4 patients) with retinal angiomatous proliferation (RAP). The mean duration from initial diagnosis to the initiation of treatment with faricimab was 39.3 ± 12.5 months. Before switching to faricimab, patients had received an average of 13.4 ± 5.8 anti-VEGF injections (the mean number of aflibercept injections: 8.5 ± 4.9), and the average injection interval was 6.8 ± 2.4 weeks. The duration since the last aflibercept injection was 5.2 ± 1.8. At baseline, the mean BCVA was 0.51 ± 0.13 logMAR, and the mean central subfield thickness (CST) was 356.2 ± 38.4 µm. The mean subfoveal choroidal thickness (SFCT) was 182.7 ± 22.8 µm before switching to faricimab. PED was present in 91.7% of eyes. Among them, 25.4% of patients had predominantly serous PED, 47.3% had predominantly fibrovascular PED, and 27.3% had purely fibrovascular PED. The mean maximum PED height and width were 309.1 ± 32.1 µm and 2327.4 ± 842.3 µm, respectively.

### 3.2. Visual and Anatomic Outcomes over 6 Months

The average follow-up period after switching to faricimab was 26.4 ± 1.9 weeks, with a mean of 2.4 ± 0.4 injections administered for 6 months. The injection interval increased significantly from 6.8 ± 2.4 weeks at baseline to 11.2 ± 1.7 weeks at month 6 (*p* < 0.05). The BCVA did not show a significant improvement 6 months after switching to faricimab (*p* = 0.150). There was a significant reduction in CST, with the mean CST decreasing to 324.5 ± 45.1 μm at 6 months after switching to faricimab (*p* = 0.020) (Table 2).

**Figure 1:**
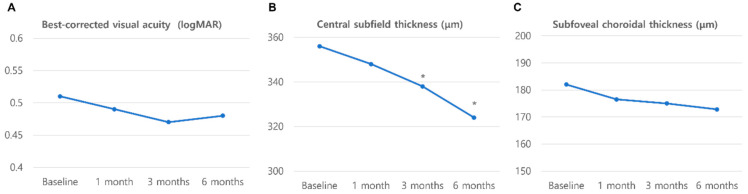
Changes in visual and anatomic outcomes before and after switching to faricimab. * *p* < 0.05.

Figure 1 shows the changes in BCVA, CST, and SFCT for 6 months. The BCVA did not change over the 6 months of faricimab treatment. The CST decreased significantly from 356.2 ± 38.4 µm before the initial treatment to 338.7 ± 49.7 µm at 3 months (*p* < 0.05) and was maintained at 324.5 ± 45.1 µm (*p* < 0.01) at 6 months. The SFCT did not significantly change throughout the 6 months.

### 3.3. Changes in PED

**Figure 2:**
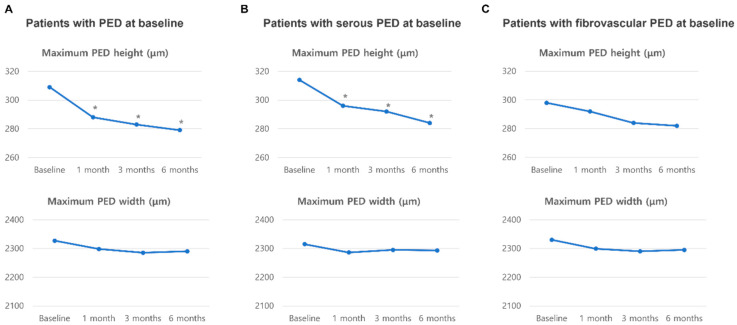
Changes in PED before and after switching to faricimab. * *p* < 0.05.

The changes in PED height and width are shown in Figure 2. The maximum PED height significantly decreased from 309.1 ± 32.1 µm at baseline to 279.0 ± 29.5 µm at six months (*p* = 0.030), with the most notable reduction occurring within the first month after switching to faricimab. For patients with predominantly serous PED at baseline, the maximum PED height significantly decreased from 314.2 ± 29.3 µm to 284.3 ± 25.4 µm by six months (*p* < 0.05). However, patients with predominantly fibrovascular PED or purely fibrovascular PED at baseline did not show a significant decrease in PED height. The PED width remained unchanged throughout the follow-up period, regardless of its type.

### 3.4. Changes in Retinal Fluid Compartment

**Figure 3:**
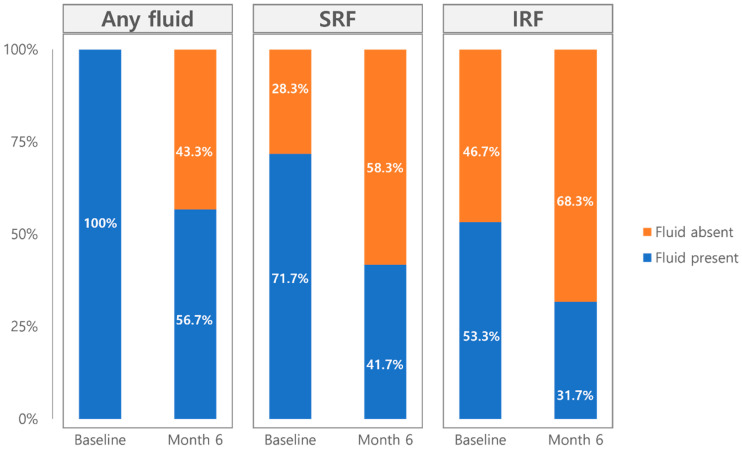
Retinal fluid compartment at baseline and month 6 after switching to faricimab.

The type of retinal fluid status improved significantly following faricimab treatment, as shown in Figure 3. At baseline, 100% of patients exhibited SRF or IRF, which decreased to 56.7% at month 6. SRF was present in 71.7% of patients at baseline and decreased to 41.7% by six months. IRF also showed improvement, decreasing from 53.3% at baseline to 31.7% at month 6. In multivariate analysis, the mean injection interval before switching (*p* = 0.030; β = 0.178) and maximum PED height (*p* = 0.048; β = −0.602) were significantly associated with the complete resolution of SRF and IRF at 6 months after switching to faricimab (Table 3).

## 4. Discussion

This study evaluated the real-world efficacy of switching to faricimab in patients with aflibercept-resistant nAMD, with a particular focus on its impact on PED characteristics. Over the six-month follow-up period, we observed stable visual acuity, significant reductions in CST and PED height, and an increase in treatment intervals, demonstrating the potential benefits of faricimab in this patient population.

We observed a significant reduction in CST following faricimab treatment. This reduction in retinal thickness was accompanied by resolution of retinal fluid, as the proportion of patients with SRF and IRF significantly decreased to 56.7% over 6 months. The ability of faricimab to stabilize the retinal microenvironment through the dual inhibition of VEGF-A and Ang-2 may contribute to these improvements by reducing vascular permeability and inflammation [10,12].

Recent studies reported that increased PED height and its fluctuations were linked to worse visual acuity outcomes and a higher incidence of SRF and IRF [18,19,20]. Therefore, evaluating absolute PED height and its variability is crucial for assessing disease activity and monitoring treatment response. Several studies have shown that PED is less responsive to anti-VEGF treatment compared to other fluid components, likely due to its composition of fibrovascular tissue and fluid [19,20]. One of the key findings of our study is that while the maximum PED height significantly decreased after switching to faricimab, the PED width remained unchanged regardless of PED type. This finding is consistent with previous reports indicating that anti-VEGF treatments primarily affect PED height, reinforcing the notion that anti-VEGF agents have a limited impact on the lateral dimensions of the detachment [20,21,22]. Thus, the timely administration of agents such as faricimab, which can influence PED dynamics, may be critical in preventing the recurrence or development of retinal fluid. The significant reduction in PED height, particularly in predominantly serous PED, suggests that faricimab effectively mitigates vascular leakage and fluid accumulation in these patients. This is supported by post hoc analyses from the TENAYA and LUCERNE trials, where faricimab’s dual inhibition of Ang-2 and VEGF-A demonstrated promising results in reducing PED [23]. By stabilizing endothelial junctions, Ang-2 inhibition helps counteract inflammatory and pro-permeability signals, while VEGF inhibition reduces fluid accumulation beneath the RPE. This combined mechanism is particularly advantageous for patients who do not respond to conventional anti-VEGF therapies, as it targets both vascular instability and permeability-related pathology.

In this study, SFCT did not change after switching to faricimab, while reductions in retinal fluid and PED were observed. Some studies suggest that long-term VEGF inhibition may lead to choroidal blood flow reduction and atrophy, potentially impacting visual prognosis [24,25]. However, the concurrent inhibition of Ang-2 by faricimab may contribute to the stabilization of the choroidal vasculature, preventing these adverse effects. By enhancing vascular stability while minimizing the risk of choroidal thinning, it may offer a more balanced and sustained therapeutic approach compared to anti-VEGF monotherapy.

Despite the anatomical benefits, BCVA remained stable without significant improvement. This finding is consistent with previous studies of treatment-resistant nAMD, where structural improvements do not always translate into immediate functional gains [14,15,16,17,18]. Factors such as prolonged disease duration, irreversible RPE damage, and the atrophy of the photoreceptor layer may limit the extent of visual recovery even when anatomical responses are favorable.

The frequent injections required for nAMD treatment pose significant challenges for both patients and healthcare systems, with reduced compliance and increased financial burden often complicating long-term management. Previous studies have reported injection interval extensions with faricimab in nAMD resistant to other anti-VEGF agents. For example, Kishi et al. noted an extension from 5.9 ± 1.5 to 7.5 ± 2.3 weeks, while Kataoka et al. reported intervals increasing from 4.4 ± 0.5 to 8.7 ± 1.7 weeks [14,18]. In our study, the interval was extended to 11.2 ± 1.7 weeks, surpassing previously reported outcomes. Differences in treatment protocols, follow-up durations, and baseline features of patients could explain the variance in outcomes.

While these findings provide valuable insights, this study has some limitations. First, this study had a retrospective design and relatively short follow-up periods. Long-term prospective studies are needed to determine whether the observed benefits are sustained over time. Second, the evaluation of OCT images was based on two-dimensional thickness (i.e., maximum PED height and width) rather than a three-dimensional volumetric assessment. However, there was a report that PED thickness measurements showed a reasonably good correlation with volume [26].

In conclusion, switching to faricimab in aflibercept-resistant nAMD patients resulted in significant anatomical improvements, particularly in PED height and CST, while maintaining stable vision and reducing treatment burden. These findings support the real-world efficacy of faricimab in nAMD resistant to aflibercept and highlight its potential for improving treatment outcomes, particularly for patients with persistent large PED or exudative activity despite prior anti-VEGF therapy.

## Figures and Tables

**Table 1:** Demographics and clinical characteristics when switching to faricimab.

	N = 60
Age (years)	71.8 ± 9.7
Gender (male/female)	36/24
Type of age-related macular degeneration (*n*, %)	
Typical neovascular age-related macular degeneration	24 (40)
Polypoidal choroidal vasculopathy	32 (53)
Retinal angiomatous proliferation	4 (7)
Time from diagnosis to first faricimab (months)	39.3 ± 12.5
Number of previous injections before switching	13.4 ± 5.8
Number of aflibercept injections before switching	8.5 ± 4.9
Injection interval before switching (weeks)	6.8 ± 2.4
Duration since the last injection (aflibercept, weeks)	5.2 ± 1.8
Best-corrected visual acuity (logMAR)	0.51 ± 0.13
Central subfield thickness (μm)	356.2 ± 38.4
Subfoveal choroidal thickness (µm)	182.7 ± 22.8
Eyes with pigment epithelial detachment (*n*, %)	55 (91.7)
Type of pigment epithelial detachment (*n*, %)	
Predominantly serous	14 (25.4)
Predominantly fibrovascular	26 (47.3)
Fibrovascular only	15 (27.3)
Maximum pigment epithelial detachment height (µm)	309.1 ± 32.1
Maximum pigment epithelial detachment width (µm)	2327.4 ± 842.3

**Table 2:** Clinical outcomes before and 6 months after switching to faricimab.

	Baseline	1 Month	3 Months	6 Months	*p*-Value
Best-corrected visual acuity (logMAR)	0.51 ± 0.13	0.49 ± 0.17	0.47 ± 0.16	0.48 ± 0.14	0.150
Central subfield thickness (μm)	356.2 ± 38.4	348.8 ± 42.1	338.7 ± 49.7	324.5 ± 45.1	0.020
Subfoveal choroidal thickness (μm)	182.7 ± 22.8	176.5 ± 28.7	175.1 ± 24.3	172.8 ± 25.9	0.072
Maximum pigment epithelial detachment height (μm)	309.1 ± 32.1	288.2 ± 26.4	283.7 ± 31.7	279.0 ± 29.5	0.030
Maximum pigment epithelial detachment width (μm)	2327.4 ± 842.3	2298.6 ± 764.2	2285.9 ± 658.3	2290.3 ± 723.8	0.068

**Table 3:** Factors associated with the complete resolution of subretinal fluid and intraretinal fluid at 6 months after switching to faricimab.

Factors	*p* *	β	95% CI
Age	0.723		
Gender	0.562		
Type of AMD	0.056		
Number of aflibercept injections before switching	0.460		
Mean injection interval before switching	0.030	0.178	0.039–0.786
Central subfield thickness (μm)	0.319		
Subfoveal choroidal thickness (μm)	0.239		
Maximum PED height (μm)	0.048	−0.602	0.340–0.090
Maximum PED width (μm)	0.649		

* Statistical analysis using binary logistic regression. AMD, age-related macular degeneration; CI, confidence interval; PED, pigment epithelial detachment.

## Data Availability

The data presented in this manuscript is not publicly accessible due to privacy restrictions.

## References

[1] Lim L.S., Mitchell P., Seddon J.M., Holz F.G., Wong T.Y. (2012). Age-related macular degeneration. Lancet.

[2] Higashijima F., Hasegawa M., Yoshimoto T., Kobayashi Y., Wakuta M., Kimura K. (2023). Molecular mechanisms of TGFβ-mediated EMT of retinal pigment epithelium in subretinal fibrosis of age-related macular degeneration. Front. Ophthalmol..

[3] Ferrante N., Ritrovato D., Bitonti R., Furneri G. (2022). Cost-effectiveness analysis of brolucizumab versus aflibercept for the treatment of neovascular age-related macular degeneration (nAMD) in Italy. BMC Health Serv. Res..

[4] Deng W., Yi C., Pan W., Liu J., Qi J., Chen J., Zhou Z., Duan Y., Ning X., Li J. (2023). Vascular Cell Adhesion Molecule-1 (VCAM-1) contributes to macular fibrosis in neovascular age-related macular degeneration through modulating macrophage functions. Immun. Ageing..

[5] Liu D., Zhang C., Zhang J., Xu G.T., Zhang J. (2023). Molecular pathogenesis of subretinal fibrosis in neovascular AMD focusing on epithelial-mesenchymal transformation of retinal pigment epithelium. Neurobiol. Dis..

[6] Moon B.H., Kim Y., Kim S.Y. (2023). Twenty Years of Anti-Vascular Endothelial Growth Factor Therapeutics in Neovascular Age-Related Macular Degeneration Treatment. Int. J. Mol. Sci..

[7] Wykoff C.C., Clark W.L., Nielsen J.S., Brill J.V., Greene L.S., Heggen C.L. (2018). Optimizing Anti-VEGF Treatment Outcomes for Patients with Neovascular Age-Related Macular Degeneration. J. Manag. Care Spec. Pharm..

[8] ElSheikh R.H., Chauhan M.Z., Sallam A.B. (2022). Current and Novel Therapeutic Approaches for Treatment of Neovascular Age-Related Macular Degeneration. Biomolecules.

[9] Cheng A.M., Joshi S., Banoub R.G., Saddemi J., Chalam K.V. (2023). Faricimab Effectively Resolves Intraretinal Fluid and Preserves Vision in Refractory, Recalcitrant, and Nonresponsive Neovascular Age-Related Macular Degeneration. Cureus.

[10] Sahni J., Dugel P.U., Patel S.S., Chittum M.E., Berger B., Del Valle Rubido M., Sadikhov S., Szczesny P., Schwab D., Nogoceke E. (2020). Safety and Efficacy of Different Doses and Regimens of Faricimab vs Ranibizumab in Neovascular Age-Related Macular Degeneration: The AVENUE Phase 2 Randomized Clinical Trial. JAMA Ophthalmol..

[11] Liberski S., Wichrowska M., Kocięcki J. (2022). Aflibercept versus Faricimab in the Treatment of Neovascular Age-Related Macular Degeneration and Diabetic Macular Edema: A Review. Int. J. Mol. Sci..

[12] Khanani A.M., Kotecha A., Chang A., Chen S.J., Chen Y., Guymer R., Heier J.S., Holz F.G., Iida T., Ives J.A. (2024). TENAYA and LUCERNE Investigators. TENAYA and LUCERNE: Two-Year Results from the Phase 3 Neovascular Age-Related Macular Degeneration Trials of Faricimab with Treat-and-Extend Dosing in Year 2. Ophthalmology.

[13] Khanani A.M., Eichenbaum D., Schlottmann P.G., Tuomi L., Sarraf D. (2018). Optimal management of pigment epithelial detachments in eyes with neovascular age-related macular degeneration. Retina.

[14] Kishi M., Miki A., Kamimura A., Okuda M., Matsumiya W., Imai H., Kusuhara S., Nakamura M. (2023). Short-Term Outcomes of Faricimab Treatment in Aflibercept-Refractory Eyes with Neovascular Age-Related Macular Degeneration. J. Clin. Med..

[15] Rush R.B., Rush S.W. (2022). Intravitreal Faricimab for Aflibercept-Resistant Neovascular Age-Related Macular Degeneration. Clin. Ophthalmol..

[16] Rush R.B. (2023). One-Year Outcomes of Faricimab Treatment for Aflibercept-Resistant Neovascular Age-Related Macular Degeneration. Clin. Ophthalmol..

[17] Goodchild C., Bailey C., Soto Hernaez J., Ahmed E., Salvatore S. (2024). Real world efficacy and durability of faricimab in patients with neovascular AMD (nAMD) who had sub-optimal response to prior anti-VEGF therapy. Eye.

[18] Kataoka K., Itagaki K., Hashiya N., Wakugawa S., Tanaka K., Nakayama M., Yamamoto A., Mukai R., Honjyo J., Maruko I. (2024). Six-month outcomes of switching from aflibercept to faricimab in refractory cases of neovascular age-related macular degeneration. Graefes Arch. Clin. Exp. Ophthalmol..

[19] Sarraf D., Khanani A.M., Sadda S.R., Chang A., Wong D.T., Kempf A.S., Saffar I., Tang S., Tadayoni R. (2024). Pigment epithelial detachment thickness and variability affects visual outcomes in patients with neovascular age-related macular degeneration. Retina.

[20] Schmidt-Erfurth U., Waldstein S.M., Deak G.G., Kundi M., Simader C. (2015). Pigment epithelial detachment followed by retinal cystoid degeneration leads to vision loss in treatment of neovascular age-related macular degeneration. Ophthalmology.

[21] Sun Z., Yang Y., Lin B., Huang Y., Zhou R., Yang C., Li Y., Huang S., Liu X. (2023). Comparative efficacy of aflibercept and ranibizumab in the treatment of age-related macular degeneration with retinal pigment epithelial detachment: A systematic review and network meta-analysis. BMC Ophthalmol..

[22] Selvam A., Singh S.R., Arora S., Patel M., Kuchhal A., Shah S., Ong J., Rasheed M.A., Manne S.R., Ibrahim M.N. (2023). Pigment epithelial detachment composition indices (PEDCI) in neovascular age-related macular degeneration. Sci. Rep..

[23] Haug S., Amador M., Kotecha A., Margaron P., Stoilov I., Tang Y. (2024). Clinical pearls from the TENAYA/LUCERNE trials of faricimab in patients with nAMD. Investig. Ophthalmol. Vis. Sci..

[24] Hoven E., Michelet J.T., Vettore M.V., Lagali N. (2025). Choroidal thickness after anti-vascular endothelial growth factor in typical neovascular age-related macular degeneration—A systematic review and meta-analysis. Surv. Ophthalmol..

[25] Fan W., Abdelfattah N.S., Uji A., Lei J., Ip M., Sadda S.R., Wykoff C.C., TREX-AMD Study Group (2018). Subfoveal choroidal thickness predicts macular atrophy in age-related macular degeneration: Results from the TREX-AMD trial. Graefes Arch. Clin. Exp. Ophthalmol..

[26] Heussen F.M., Ouyang Y., Sadda S.R., Walsh A.C. (2011). Simple estimation of clinically relevant lesion volumes using spectral domain-optical coherence tomography in neovascular age-related macular degeneration. Investig. Ophthalmol. Vis. Sci..

